# Genome-wide association and integrative analyses of relative handgrip strength identify polygenic determinants of gastrointestinal disorder susceptibility

**DOI:** 10.1186/s12876-026-04624-9

**Published:** 2026-01-27

**Authors:** Pan Jiang, Yanfei Fang, Zhengye Liu, Hanze Du, Xiaoyin Bai, Haotian Chen, Jiarui Mi

**Affiliations:** 1 Department of Gastroenterology, Sir Run Run Shaw Hospital, Zhejiang University School of Medicine, Hangzhou, Zhejiang 310016 China; 2 Department of Plastic and Aesthetic Center, The First Affiliated Hospital, Zhejiang University School of Medicine, Hangzhou, Zhejiang 310016 China; 3 Department of Endocrinology, Key Laboratory of Endocrinology of National Health Commission, Translation Medicine Centre, Peking Union Medical College Hospital, Peking Union Medical College, Chinese Academy of Medical Sciences, Beijing, 100730 China; 4 Department of Gastroenterology, Peking Union Medical College Hospital, Peking Union Medical College and Chinese Academy of Medical Sciences, Beijing, 100730 China

**Keywords:** Relative hand grip strength, Genome-wide association study, Polygenic risk score, Hernia, Diverticular intestine

## Abstract

**Background:**

Hand grip strength is a crucial indicator of muscle strength and quality. Yet, there remain significant knowledge gaps in our understanding of the genetic factors that influence hand grip strength and its impact on digestive disorders.

**Methods:**

This study employed data from the UK Biobank, comprising 405,394 individuals of European ancestry, who underwent assessments of both left- and right- hand grip strength at baseline. We calculated the relative hand grip strength (RHGS), defined as the average hand grip strength adjusted for body mass index (BMI). Subsequently, we performed genome-wide association study (GWAS) to identify RHGS-linked variants and genes. We evaluated RHGS’s impact on digestive disorders using linkage disequilibrium score regression (LDSC), Mendelian randomization (MR), Polygenic risk score (PRS), regression models, and interaction analyses.

**Results:**

GWAS of 405,394 Europeans identified 1,111 independent SNPs across 226 autosomal loci and 407 genes associated with RHGS. TWAS with skeletal-muscle eQTLs prioritized genes, including *L3MBTL3*, *CEP192* and *NUCKS1*, highly expressed in type I/II myocytes and mesenchymal cells. LDSC revealed significant negative genetic correlations between RHGS and abdominal hernia (rg = -0.121, *p* = 5.00 × 10⁻⁴), diaphragmatic hernia (rg = -0.155, *p* = 6.33 × 10⁻⁶) and diverticular intestine (rg = -0.141, *p* = 3.85 × 10⁻¹⁰). MR indicated that one-unit higher genetically predicted RHGS reduced odds of diaphragmatic hernia (OR = 0.45, 95% CI 0.25–0.82), diverticular intestine (OR = 0.42, 0.26–0.66), NAFLD (OR = 0.49, 0.29–0.83) and peptic ulcer (OR = 0.54, 0.31–0.97). Each standard-deviation increase in RHGS PRS was associated with lower risks of abdominal hernia (OR = 0.98), diaphragmatic hernia (OR = 0.96) and diverticular intestine (OR = 0.98) after full adjustment. Gene-environment analyses showed diabetes, hyperlipidemia and smoking attenuated the protective effects from elevated RHGS, whereas cardioprotective diet and higher fiber intake showed synergistic protection on abdominal hernia, diaphragmatic hernia and diverticular intestine.

**Conclusion:**

We identified multiple RHGS-associated loci enriched in skeletal muscle. Genetically high RHGS inversely correlated with risks of abdominal hernia, diaphragmatic hernia and diverticular intestine. RHGS polygenic risk score enables risk stratification, and its effects are modulated by diet and common comorbidities.

**Graphical Abstract:**

The schematics of the study pipeline in the investigation of RHGS GWAS and the follow-up post-GWAS analyses for clinical relevance. RHGS: relative hand grip strength; GWAS: genome-wide association study.

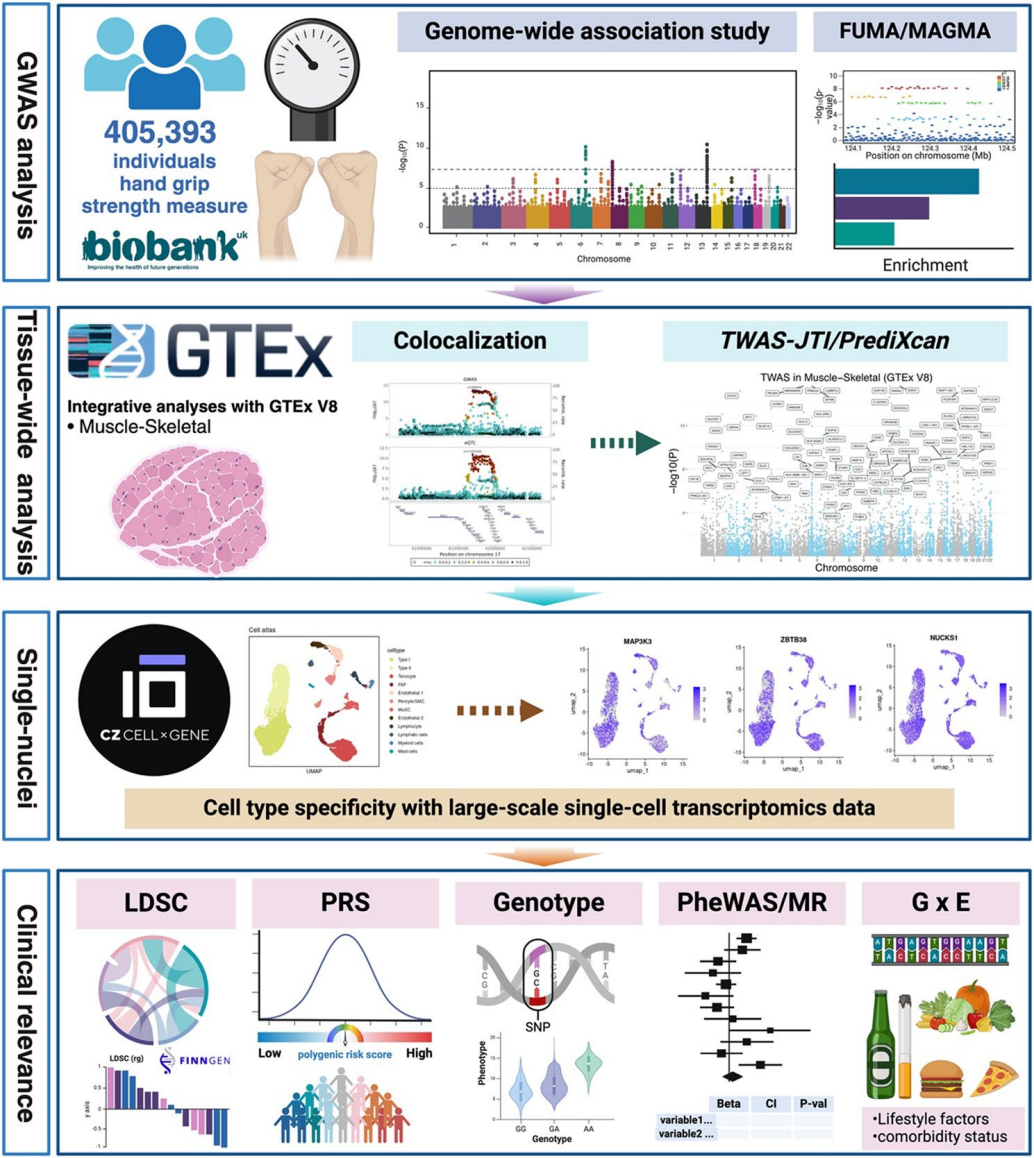

**Supplementary Information:**

The online version contains supplementary material available at 10.1186/s12876-026-04624-9.

## Highlights

1. GWAS revealed hundreds of independent SNPs and genes linked to RHGS.

2. TWAS and colocalization prioritized multiple skeletal muscle related-genes associated with RHGS.

3. RHGS showed significant negative genetic correlations and inverse causation with many digestive disorders.

4. Polygenic risk score for RHGS predicted decreased risk of abdominal hernia, diaphragmatic hernia and diverticular intestine.

5. Lifestyle factors and comorbidities might modify genetic associations between RHGS and digestive diseases.

**Supplementary Information:**

The online version contains supplementary material available at 10.1186/s12876-026-04624-9.

## Introduction

Hand grip strength (HGS) refers to the force exerted by hand muscles, serving as an essential indicator for the assessment of muscle strength, muscle quality and overall health. Measuring grip strength is simple and non-invasive, making it widely used in clinical and epidemiological studies. A decline in HGS is associated with various health issues, such as cardiovascular disease, diabetes, cognitive decline, and increased all-cause mortality [1]. It is also used to reflect changes in nutritional status [2]. Common factors influencing hand grip strength include age, gender, body mass index (BMI), body composition, lifestyle factors, and health status. Studies show that HGS is a surrogate marker of aging, which decreases with age. Higher BMI and better body compositions are often associated with stronger HGS [3, 4]. HGS is commonly measured using a dynamometer. To ensure accuracy, multiple measurements are taken and averaged, though variations in equipment and methods can affect results [3, 5]. The impact of HGS on the onset and progression of diseases has been confirmed in multiple studies. Higher HGS is typically associated with lower cardiovascular disease risk, better cognitive function and lower all-cause mortality [6, 7]. Additionally, HGS has been found to be linked to the incidence and mortality rates of various cancers [8, 9]. Apart from environmental and lifestyle factors, recent studies have also showed the HGS is largely determined by genetic factors [10, 11].

Relative hand grip strength (RHGS) refers to the ratio of hand grip strength to an individual’s BMI which better reflects an individual’s muscular function due to the consideration of body type differences. Although genetic variants from prior studies in HGS have been revealed by genome-wide association study (GWAS), due to the limitation in sample size, the ignorance of handedness and pleiotropic effects on body composition, the genetic architecture of RHGS still require further investigation. Moreover, the evidence for the prognostic value of RHGS in digestive health is still lacking. Emerging cohort evidence links weaker grip to incident gastrointestinal disease: in 393 606 adults followed for 12 years every 5-kg higher HGS was associated with 6–20% lower risk of 16 GI outcomes, including diverticular intestine (HR 0.87) and abdominal hernia (HR 0.82) [12]. Mechanistically, declining muscle mass fosters chronic low-grade inflammation (IL-6, TNF-α), insulin resistance and depletion of myokines that maintain intestinal wall integrity, collectively predisposing to mucosal vulnerability, collagen cross-link defects and raised intraluminal pressure, the pathogenic processes central to herniation and diverticula formation [13]. RHGS, by indexing muscle power relative to body size, may therefore capture the component of GI risk attributable to compromised muscular and metabolic support of the gut.

To elucidate the genetic architecture of RHGS and its clinical relevance, we conducted a GWAS of RHGS, which is defined as the average of left- and right-hand grip strength normalized by BMI, in 405,394 UK Biobank participants of European ancestry. We identified susceptibility variants and prioritized skeletal muscle-effector genes by integrating post-GWAS pipelines including colocalization, TWAS, functional enrichment and single-nucleus transcriptomics. We used LD score regression and mendelian randomization to reveal the polygenicity across different disorders and constructed a polygenic risk score (PRS) for risk stratification. Finally, we performed gene-environment interaction analyses to investigate the synergistic effects between lifestyle habits or comorbidities and RHGS PRS on the risk of certain digestive disorders.

## Method

### Study cohort and GWAS analysis

The UK Biobank initiative is a prospective longitudinal study that has enrolled over 500,000 participants aged 40 to 69 years across the United Kingdom [14]. The genotyping process was executed in two phases, utilizing the Affymetrix UK BiLEVE Axiom chip and the Affymetrix UK Biobank Axiom array. Our GWAS was performed in 405,393 individuals of European ancestry with both left- and right-hand grip strength measurements at baseline. We excluded those with ambiguous sex information, missing genetic data, or outliers in heterozygosity. Population stratification was addressed through Principal Component Analysis (PCA), removing individuals who exceeded 5 standard deviations from the mean on the first two principal components (PC1 and PC2). Additionally, individuals demonstrating high kinship were excluded based on a kinship coefficient threshold of > 0.0884. The GWAS was conducted using a linear mixed model (LMM) implemented in the “BOLT-LMM” software based on the RHGS (a continuous variable), adjusting for the first 10 principal components, genotype measurement batch, age at assessment, assessment center, and BMI. Individual-level UK Biobank data are available to approved researchers through the UK Biobank Access Management System (https://www.ukbiobank.ac.uk/). SNP-heritability (h²SNP) of RHGS was estimated with LDSC and GCTA-GREML. For GCTA, we used 50 000 unrelated EUR participants (kinship < 0.025) and 1.1 million QC-filtered SNPs to build a GRM; RHGS was regressed on the GRM while adjusting for sex, age, age², BMI, assessment centre and 20 PCs. Estimates were transformed to the liability scale assuming 0.5 prevalence.

### Enrichment, TWAS, Co-localization and single-nuclei RNA-seq

Enrichment analyses were conducted utilizing the “ClusterProfiler” and “goprofiler2” tools. The PrediXcan and JTI methodologies were employed in the transcriptome-wide association study (TWAS) [15]. The gene expression data utilized in the analyses were derived from the GTEx V8 datasets. For the primary analysis, expression quantitative trait loci (eQTL) data from skeletal muscle tissue were used to identify SNPs that may influence gene transcription levels within 1 Mb region in a tissue-specific context. Linkage disequilibrium (LD) information for plotting was obtained from the 1000 Genomes Project Phase 3 dataset. To elucidate the key cell types within skeletal muscle that express the selected genes, we analyzed publicly available single-nuclei RNA sequencing data from a recent study by Lai et al. [16]. The DNBelab C Series Single-Cell Library Prep Set platform was employed for transcriptional profiling. To mitigate pleiotropy/LD noise, TWAS hits were fine-mapped with FOCUS v0.7 (GTEx-muscle LD; ±500 kb).

### LD score regression and Mendelian randomization

We extracted summary statistics of 546 common disorders from the FinnGen R10 datasets and evaluated their genetic correlations with RHGS using the cross-trait LDSC regression method [17]. FinnGen R10 summary statistics were obtained from the project’s public repository (https://finngen.gitbook.io/documentation/r10/data-download). The complete set of files offered through the batch-download link was retrieved without further filtering. This method employs a weighted linear regression framework to estimate genetic correlations by regressing the product of Z-scores from each disease pair against the LD scores of SNPs across the genome. To ascertain statistically significant correlations, we applied a Benjamini-Hochberg-corrected p-value threshold. Additionally, to investigate the causal relationships between RHGS and systemic disorders, we utilized the two-sample Mendelian randomization (MR) approach, implemented via the “TwoSampleMR” R package, with instrumental variables subjected to clumping at an R² threshold of 0.001 [18–20].

### Polygenic risk score and statistical analyses

A polygenic risk score (PRS) was developed based on the leading SNPs identified in GWAS following the clumping criteria (*P* < 5 × 10^− 8^; r² < 0.001) to estimate the genetically predisposed levels of RHGS [21]. The detailed methodology for PRS construction has been previously documented in earlier studies. Participants were stratified into low, medium, and high genetic risk categories according to the tertile levels of the PRS. Individuals were classified according to the RHGS PRS. Logistic regression and Cox proportional hazards regression models were employed to estimate the odds ratios (ORs) and hazard ratios (HRs) with 95% confidence intervals (CIs), using the lowest tertile as the reference group. The multivariable-adjusted models (Model 2 to Model 5) accounted for baseline age, sex, ethnicity, townsend deprivation index (TDI), BMI, type 2 diabetes, and hyperlipidemia. To explore gene-environment interactions, we assessed both multiplicative and additive interactions between RHGS and various lifestyle factors. Additive interactions were evaluated using the relative excess risk due to interaction, attributable proportion due to interaction, and synergy index. Joint associations were analyzed by categorizing individuals based on the combination of RHGS categorical groups and selected lifestyle factors. We calculated a cardioprotective-diet score that aligns with American Heart Association guidelines for cardiometabolic health [29]. Food-frequency questionnaire responses were scored against seven pre-defined targets: ≥ 3 daily servings each of fruits, vegetables and whole grains; ≥ 2 weekly servings of fish; ≤ 2 daily servings of refined grains; ≤ 2 weekly servings of unprocessed red meats; and ≤ 1 weekly serving of processed meats. One point was awarded for each target met, giving a 0–7 scale on which higher values indicate greater adherence. To ensure balanced group sizes for subsequent analyses, the continuous score was collapsed into five ordinal categories: 0–1, 2, 3, 4 and 5–7 [22]. The ICD codes for diseases of interest were shown in Supplementary Table 1. All statistical tests were two-sided, with a p-value threshold of < 0.05 considered statistically significant except GWAS and TWAS (using Bonferroni correction). All analyses were conducted using R software version 4.2.3.

## Results

### GWAS of RHGS

**Fig. 1:**
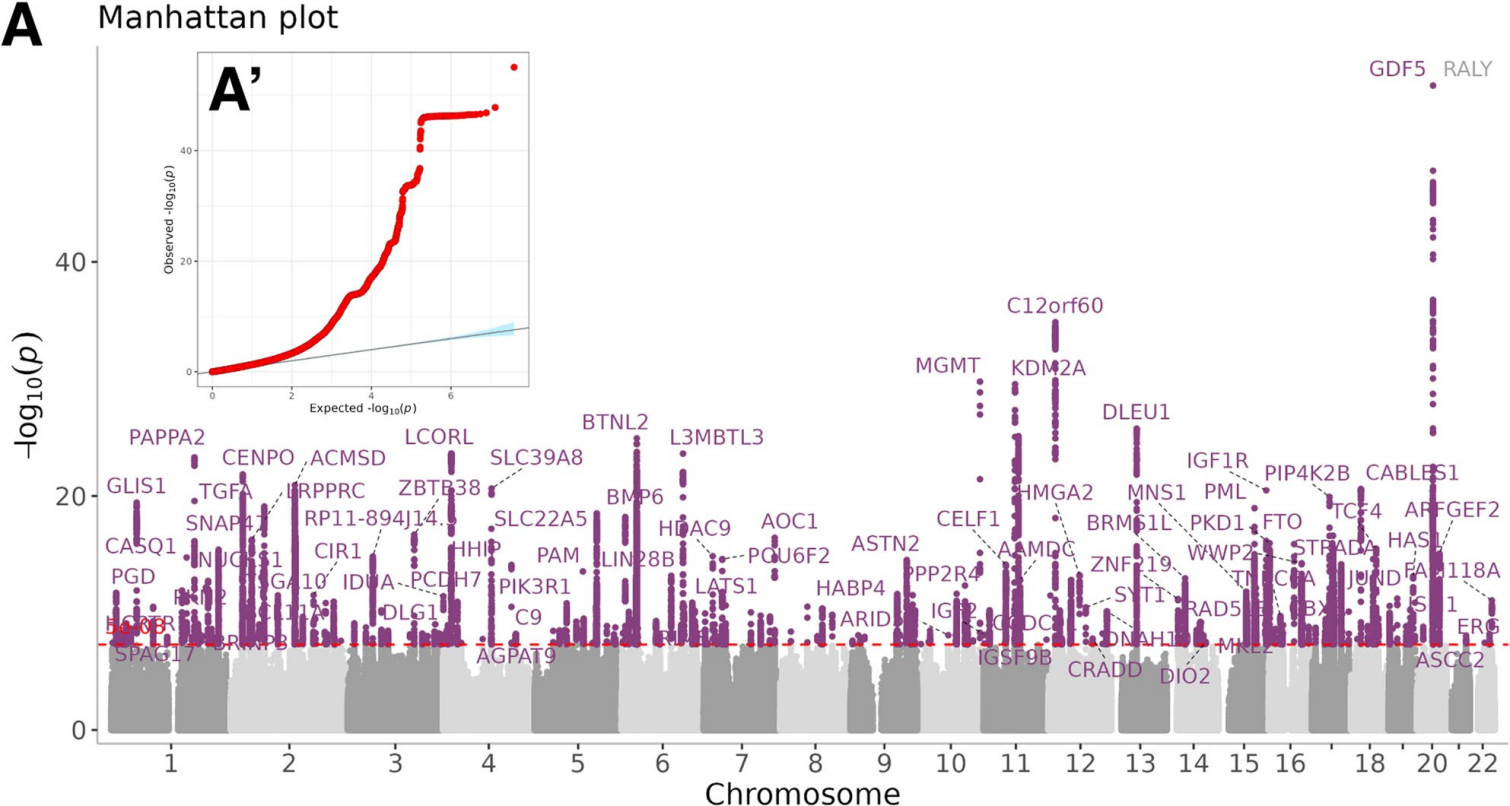
GWAS analyses of RHGS in European population from UK Biobank **A**. Manhattan plot for the *p-values* of all variants. The purple dash line indicates the genome-wide significance level of 5 × 10^− 8^. **A’.** Q-Q plot for the GWAS analyses. RHGS: relative hand grip strength. GWAS: genome-wide association study

Our GWAS identified a total of 226 genomic loci comprising 1,111 independent SNPs located on the autosomes (Fig. 1, Supplementary Tables 2–6). A total of 407 genes were successfully mapped and annotated using FUMA. The most significantly associated SNPs were predominantly found in intergenic and intronic regions, while only 0.5% to 2% of the SNPs were situated in untranslated regions, exons, and downstream regions of protein-coding genes. Functional enrichment analyses indicated that these significant loci are implicated in various biological processes, including the “regulation of primary metabolic processes” and “skeletal muscle development” (Supplementary Tables 7 and 8, Supplementary Fig. 1). LD score regression estimated the SNP-heritability of RHGS at h² = 0.18 (SE 0.01), and GCTA-GREML in an independent 50,000 subset yielded a concordant h² = 0.16 (SE 0.02), indicating that ~ 16–18% of the variance in RHGS is attributable to common variants.

### TWAS, Co-localization, and single nuclei RNA-seq mapping in skeletal muscle

**Fig. 2:**
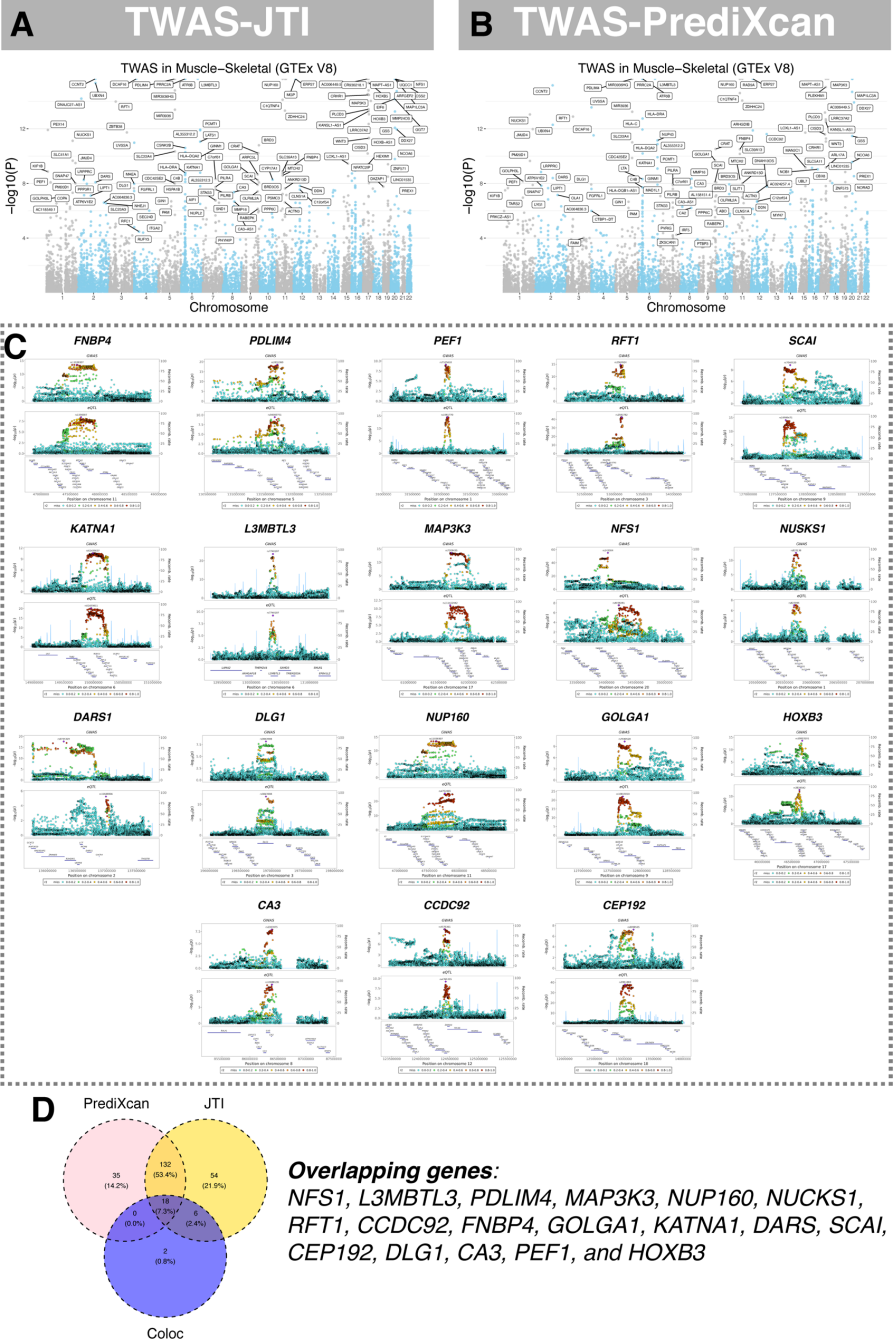
Transcriptome-wide association analyses and genetic colocalization study. **A **and** B** Manhattan plots show the significant transcript from TWAS JTI and PrediXcan. **C **Regional plots showing the top 10 colocalization with highest posterior probability between RHGS GWAS and candidate gene and loci. RHGS: relative hand grip strength. **D **Venn diagram showing the overlapping genes among colocalization and two TWAS methods in skeletal muscle. RHGS: relative handgrip strength; GWAS: genome-wide association study; TWAS: transcriptome-wide association study

**Fig. 3:**
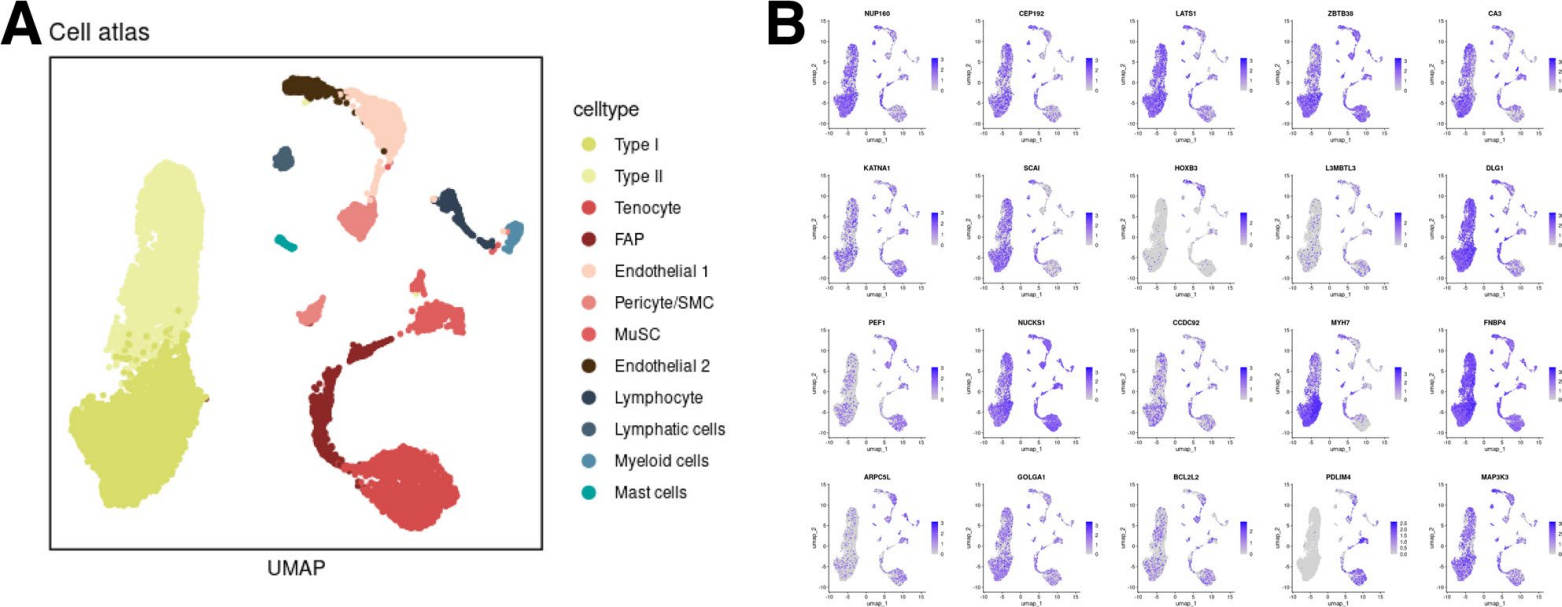
Integrative analyses with single nuclei RNA-seq gene mapping from human tissue in skeletal muscle. **A** Cell type annotation using UMAP. **B** Featureplot demonstrating the expression profiles of candidate genes in different cell types in the skeletal muscle. UMAP: Uniform Manifold Approximation and Projection

The tissue specificity analyses were shown in Supplementary Table 9. To pinpoint genes that regulate skeletal-muscle RHGS, we first conducted Bayesian colocalization between GWAS and skeletal muscle eQTL signals; this highlighted 24 candidate polymorphisms and their cognate genes (Supplementary Table 10). We then performed summary-statistic TWAS in the same tissue (Fig. 2A-B, Supplementary Tables 11 and 12), identifying > 180 genes whose expression associates with RHGS after Bonferroni correction (*p* < 3.694 × 10⁻⁶). A Venn diagram (Fig. 2D) shows the overlapping results among two TWAS methods and colocalization results in the skeletal muscle. In total 18 genes (*NFS1*, *L3MBTL3*, *PDLIM4*, *MAP3K3*, *NUP160*, *NUCKS1*, *RFT1*, *CCDC92*, *FNBP4*, *GOLGA1*, *KATNA1*, *DARS*, *SCAI*, *CEP192*, *DLG1*, *CA3*, *PEF1*, and *HOXB3*) were shared by all three approaches, indicating high-confidence using different approaches in dictating handgrip strength in skeletal muscle. The FOCUS finemapping results were shown in Supplementary Table 14 Single-nucleus transcriptomics confirmed that the majority of these genes are highly expressed in type I/II myocytes, mesenchymal cells (endothelial cells and pericytes) and inflammatory infiltrates (Fig. 3).

### Phenome-wide LD score regression and Mendelian randomization

**Fig. 4:**
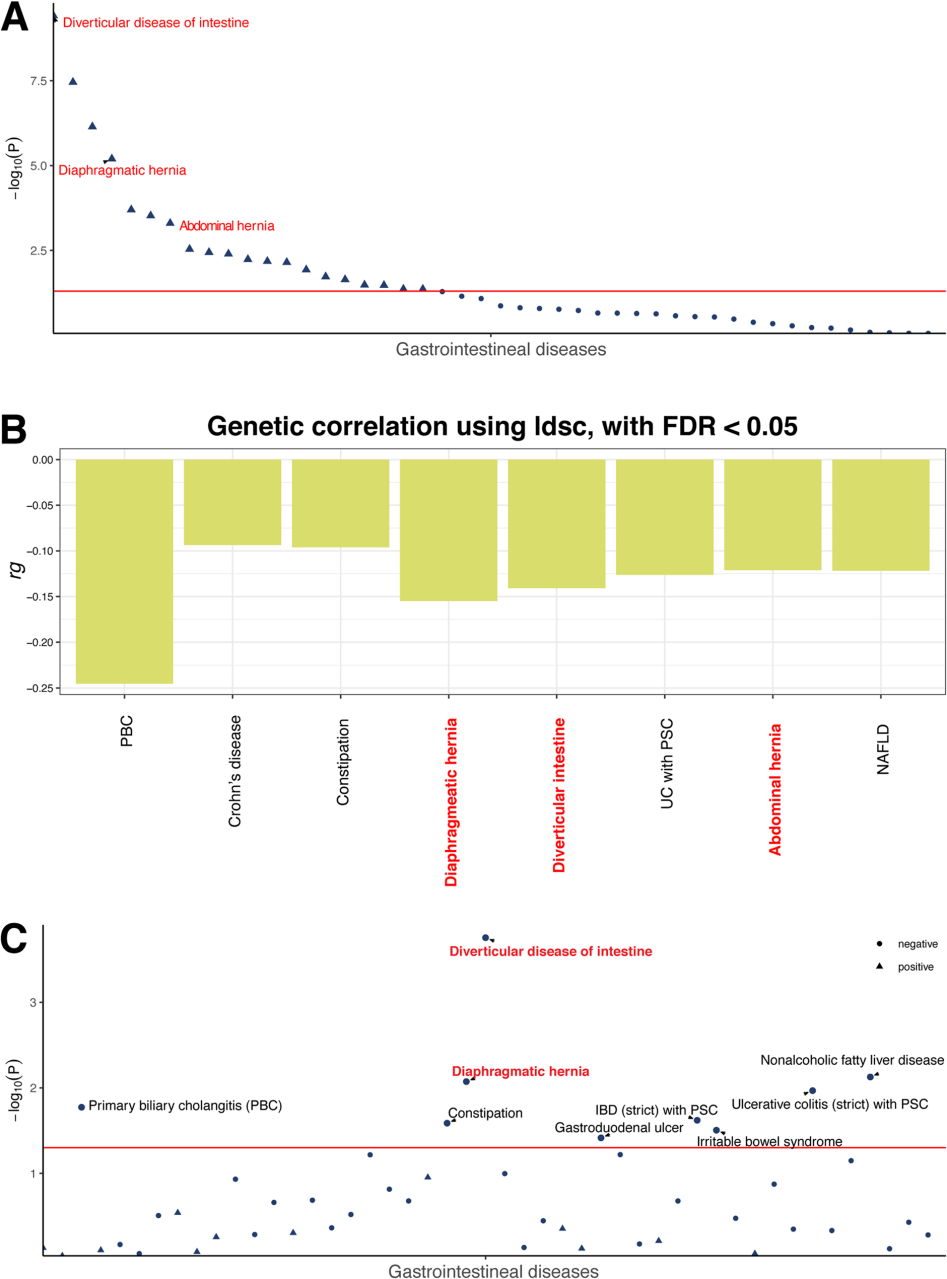
Phenome-wide LD score regression and mendelian randomization of RHGS. **A** The *p-values* of genetic correlations 546 diseases from FinnGen project (R10) with RHGS GWAS. The gray dash line is p-value = 0.05. The dotplot showing the genetic correlation significance between the RHGS and major gastrointestinal diseases recorded in FinnGen R10. Diseases with FDR < 0.05 are represented by triangles; diseases with *p-value* ≥ 0.05 are represented by dots. **B** The regression coefficient of significant genetic correlations with RHGS. The colors denote different disease categories. The gray dash line is *p-value* = 0.05. The barplot showing regression coefficient of significant genetic (rg) correlations between RHGS and major gastrointestinal diseases with FDR < 0.05. The rg < 0 indicates negative genetic correlation, while rg > 0 indicates positive genetic correlation. **C** The dotplot showing the causal inferences using MR IVW method of the RHGS on the risks of major gastrointestinal diseases recorded in FinnGen R10. Diseases with *p-value* < 0.05 are represented by triangles; diseases with *p-value* ≥ 0.05 are represented by dots. The gray dash line is *p-value* = 0.05. LD: Linkage disequilibrium; FDR: False discovery rate; CD: Crohn’s disease; PBC: Primary biliary cholangitis; PSC: Primary sclerosing cholangitis; UC: Ulcerative colitis; NAFLD: non-alcoholic fatty liver disease; MR: Mendelian randomization, IVW: Inverse variance weighted

Using LD score regression, we observed significant negative genetic correlations between genetically determined RHGS and abdominal hernia (rg = −0.121, *p* = 5.00E-04), diaphragmatic hernia (rg = −0.155, *p* = 6.33E-06) and diverticular intestine (rg = −0.1409, *p* = 3.85E-10) (Fig. 4A and B, Supplementary Table 14). Non-alcoholic fatty liver disease (NAFLD) likewise showed a nominally inverse correlation (rg = −0.122, *p* = 7.15E-07), whereas duodenal ulcer (rg = −0.198, *p* = 0.0066, FDR = 0.080) and peptic ulcer (rg = −0.1193, *p* = 0.023, FDR = 0.180) exhibited weak, non-significant trends after FDR correction. Mendelian randomization analyses indicated that a one-unit increase in genetically predicted RHGS was associated with lower odds of diaphragmatic hernia (OR = 0.45, 95% CI 0.25–0.82, *p* = 0.008), diverticular intestine (OR = 0.42, 95% CI 0.26–0.66, *p* = 1.76E-4), NAFLD (OR = 0.49, 95% CI 0.29–0.83, *p* = 0.007), and peptic ulcer (OR = 0.54, 95% CI 0.31–0.97, *p* = 0.038). While not reaching statistical significance, negative trends were observed in abdominal hernia (OR = 0.52, 95% CI 0.26–1.06, *p* = 0.071) and duodenal ulcer (OR = 0.44, 95% CI 0.16–1.17, *p* = 0.101) (Fig. 4C and Supplementary Tables 15–19). Notably, for diverticular intestine, the weighted-median OR was 0.36 (95% CI 0.22–0.59, *p* = 3.9E-5) and the MR-Egger was 0.14 ([95% CI 0.03–0.76, *p* = 0.024]), preserving nominal significance (*p* < 0.05).

### Regression analyses using RHGS PRS

**Fig. 5:**
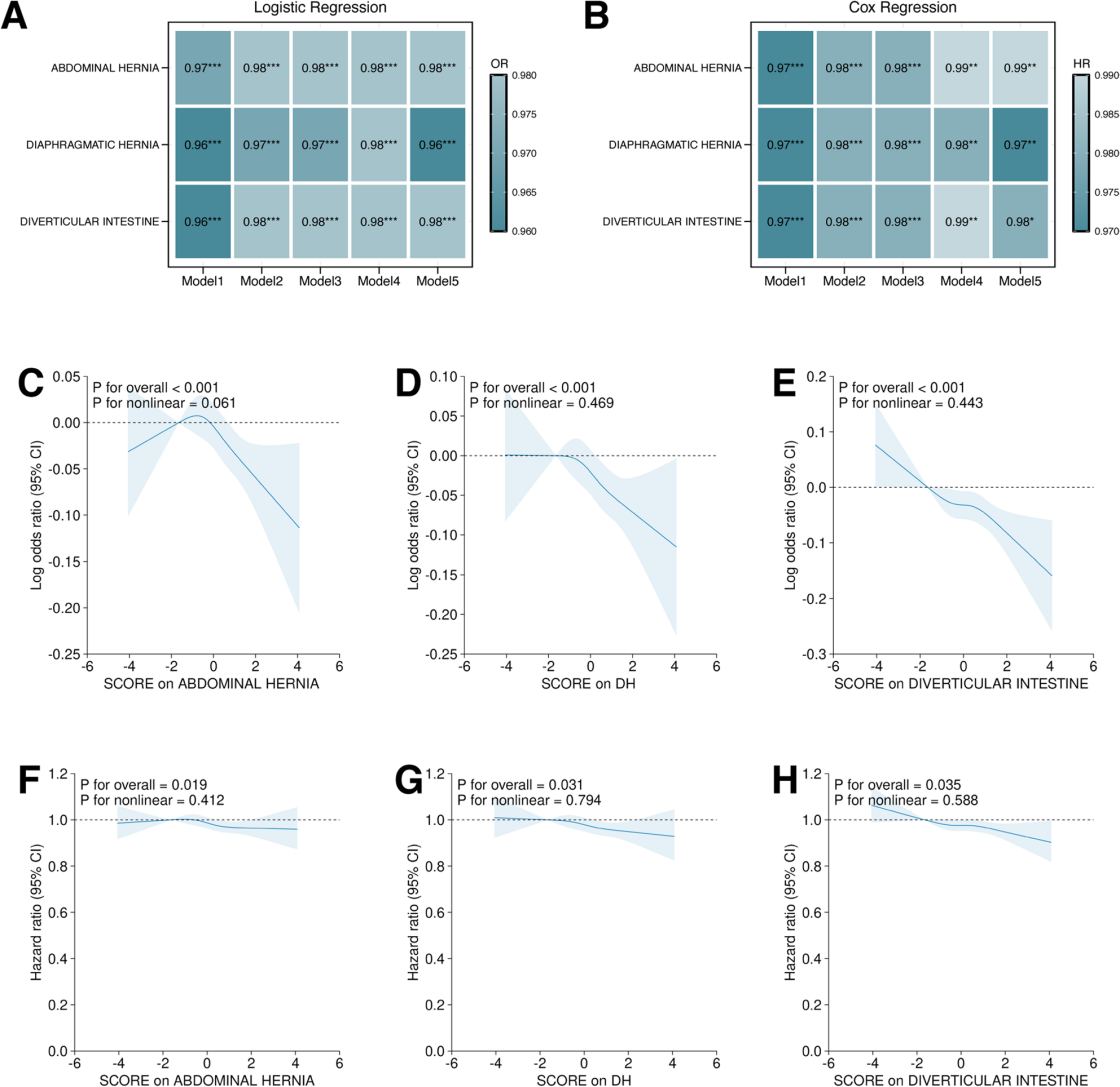
Association of polygenic risk score of RHGS on the risk of abdominal hernia, diaphragmatic hernia and diverticular intestine using regression models and restrictive cubic spline analyses. **A **and** B** Heatmap showing the logistic regression (**A**) and Cox regression (**B**) results of the polygenic risk score of RHGS (RHGS PRS) on abdominal hernia, diaphragmatic hernia and diverticular intestine. The model 1: raw model without adjustment (model 1); the model 2: multi-adjusted model with common covariates including age, sex, BMI, townsend deprivation index, smoking status, alcohol consumption, education, ethnicity and albumin levels; the model 3: with further adjustment of urate levels, diabetes, and hyperlipidemia on top of the model 2; the model 4: further adjusted model with physical activity and waist-to-height ratio on top of the model 3; the model 5: further adjusted model with cardioprotective diet on top of the model 4. **C-E** Restrictive cubic spline illustrating the potential linearity between RHGS PRS and abdominal hernia (**C**), diaphragmatic hernia (**D**), and diverticular inteatine (**E**) risks using logistic regression. **F-H** Restrictive cubic spline illustrating the potential linearity between RHGS PRS and abdominal hernia (**F**), diaphragmatic hernia (**G**), and diverticular intestine (**H**) risks using Cox regression. DH: Diaphragmatic hernia; OR: odds ratio; HR: hazards ratio

Using the RHGS PRS derived from our GWAS (P < 5 × 10⁻⁸, R² < 0.001), we performed stepwise logistic and Cox regression models across the entire cohort to test genetically driven RHGS levels for association with major hernia and diverticular disorders (Table 1). After full adjustment, each one-standard-deviation increase in the RHGS PRS was associated with reduced risks of abdominal hernia (OR = 0.98, 95% CI 0.97–0.99, *p *< 0.001), diaphragmatic hernia (OR = 0.96, 95% CI 0.95–0.97, p < 0.001) and diverticular intestine (OR = 0.98, 95% CI 0.97–0.99, p < 0.001) (Fig. 5A and B, Supplementary Table 20). Restricted cubic splines revealed significant dose-response relationships between RHGS PRS and all three outcomes (p overall < 0.05), with no evidence of departure from linearity (p non-linearity > 0.05; Fig. 5C-E). These findings were consistent across sensitivity analyses using Cox regression and RHGS measures (Figs. 5F-H, Supplementary Tables 21–23). For duodenal ulcer, gastric ulcer and peptic ulcer, both logistic and Cox regression pointed toward an inverse association; however, statistical significance was not consistently achieved. In contrast, other diseases including NAFLD, PBC, PSC, exhibited neither a consistent direction of effect nor statistical significance (Supplementary Fig. 2–4, Supplementary Tables 21–23). Lastly, to address potential sample-overlap bias, we repeated the GWAS in a 70% discovery subset. Association testing for the three major GI phenotypes was confined to the remaining 30% validation set (Supplementary Fig. 5 A and A’). It confirmed that the genetically elevated RHGS PRS remained significantly protective against abdominal hernia, diaphragmatic hernia and diverticular disease after full adjustment (Supplementary Figure. 5B and C, Supplementary Tables 24 and 25).

**Table 1:** Baseline characteristics

		Female (*N* = 215950)						Male (*N* = 185619)			
Name		Levels	Q1 (*N* = 119967)	Q2 (*N* = 79266)	Q3 (*N* = 16717)		*p*	Q1 (*N* = 13890)	Q2 (*N* = 54590)	Q3 (*N* = 117139)	*p*
BMI		Mean ± SD	29.2 ± 5.4	24.9 ± 3.3	22.2 ± 2.4		< 0.001	32.3 ± 5.9	29.5 ± 4.1	26.5 ± 3.3	< 0.001
Weight		Mean ± SD	75.7 ± 15.2	67.0 ± 10.4	62.0 ± 8.4		< 0.001	96.1 ± 19.8	89.5 ± 14.9	83.1 ± 12.2	< 0.001
age		Mean ± SD	58.3 ± 7.5	54.7 ± 7.9	50.5 ± 7.3		< 0.001	59.4 ± 7.5	58.6 ± 7.8	55.5 ± 8.2	< 0.001
Ethnics		European	109,263 (91.1%)	72,691 (91.7%)	14,977 (89.6%)		< 0.001	12,230 (88%)	49,768 (91.2%)	108,450 (92.6%)	< 0.001
		non-European	10,704 (8.9%)	6575 (8.3%)	1740 (10.4%)			1660 (12%)	4822 (8.8%)	8689 (7.4%)	
alcohol		frequent alcohol intake	38,024 (31.7%)	33,600 (42.4%)	7941 (47.5%)		< 0.001	5449 (39.2%)	26,783 (49.1%)	63,846 (54.5%)	< 0.001
		infrequent or no alcohol intake	81,943 (68.3%)	45,666 (57.6%)	8776 (52.5%)			8441 (60.8%)	27,807 (50.9%)	53,293 (45.5%)	
smoking		Current	10,106 (8.4%)	7416 (9.4%)	1746 (10.4%)		< 0.001	1728 (12.4%)	6463 (11.8%)	14,912 (12.7%)	< 0.001
		Never	70,778 (59%)	47,365 (59.8%)	10,369 (62%)			6029 (43.4%)	24,767 (45.4%)	60,075 (51.3%)	
		Previous	39,083 (32.6%)	24,485 (30.9%)	4602 (27.5%)			6133 (44.2%)	23,360 (42.8%)	42,152 (36%)	
Triglycerides		Mean ± SD	1.7 ± 0.9	1.4 ± 0.8	1.1 ± 0.6		< 0.001	2.2 ± 1.2	2.1 ± 1.2	1.9 ± 1.1	< 0.001
HbA1C		Mean ± SD	36.7 ± 6.7	34.8 ± 4.7	33.7 ± 3.9		< 0.001	40.5 ± 10.9	37.7 ± 8.4	35.4 ± 6.2	< 0.001
CRP		Mean ± SD	3.4 ± 4.8	1.9 ± 3.5	1.3 ± 2.8		< 0.001	4.0 ± 5.6	2.9 ± 4.6	2.1 ± 3.9	< 0.001
Townsend_deprivation_index		Mean ± SD	−1.2 ± 3.1	−1.6 ± 2.9	−1.6 ± 2.9		< 0.001	−0.1 ± 3.5	−1.1 ± 3.2	−1.6 ± 3.0	< 0.001
DmT2_all		No	107,249 (89.4%)	76,951 (97.1%)	16,569 (99.1%)		< 0.001	9296 (66.9%)	44,446 (81.4%)	108,872 (92.9%)	< 0.001
		Yes	12,718 (10.6%)	2315 (2.9%)	148 (0.9%)			4594 (33.1%)	10,144 (18.6%)	8267 (7.1%)	
HyperLip_all		No	88,439 (73.7%)	67,801 (85.5%)	15,529 (92.9%)		< 0.001	7264 (52.3%)	33,464 (61.3%)	87,144 (74.4%)	< 0.001
		Yes	31,528 (26.3%)	11,465 (14.5%)	1188 (7.1%)			6626 (47.7%)	21,126 (38.7%)	29,995 (25.6%)	
SCORE		Mean ± SD	−1.1 ± 0.5	−0.0 ± 0.2	0.8 ± 0.3		< 0.001	−0.9 ± 0.5	0.1 ± 0.2	1.2 ± 0.5	< 0.001
FI_score		Mean ± SD	0.1 ± 0.1	0.1 ± 0.1	0.1 ± 0.1		< 0.001	0.2 ± 0.1	0.1 ± 0.1	0.1 ± 0.1	< 0.001
MetS_Score		Mean ± SD	0.3 ± 0.8	−0.3 ± 0.7	−0.7 ± 0.6		< 0.001	1.4 ± 0.9	1.1 ± 0.8	0.7 ± 0.8	< 0.001
cci		Mean ± SD	0.3 ± 0.8	0.2 ± 0.6	0.1 ± 0.5		< 0.001	0.6 ± 1.1	0.3 ± 0.8	0.2 ± 0.6	< 0.001
metabolic_status_category		Healthy Metabolism	53,285 (44.4%)	50,740 (64%)	12,865 (77%)		< 0.001	2807 (20.2%)	15,574 (28.5%)	50,323 (43%)	< 0.001
		Unhealthy Metabolism	61,976 (51.7%)	24,515 (30.9%)	2697 (16.1%)			10,746 (77.4%)	37,239 (68.2%)	61,566 (52.6%)	
		Unknown	4706 (3.9%)	4011 (5.1%)	1155 (6.9%)			337 (2.4%)	1777 (3.3%)	5250 (4.5%)	
INFLA_score		Mean ± SD	−0.1 ± 6.6	−2.2 ± 6.3	−3.4 ± 6.2		< 0.001	0.5 ± 6.6	−1.1 ± 6.4	−2.6 ± 6.3	< 0.001
PA_category		High	30,876 (25.7%)	26,872 (33.9%)	6780 (40.6%)		< 0.001	3078 (22.2%)	17,029 (31.2%)	45,882 (39.2%)	< 0.001
		Low	18,194 (15.2%)	9626 (12.1%)	1748 (10.5%)			3191 (23%)	9479 (17.4%)	16,484 (14.1%)	
		Moderate	37,616 (31.4%)	27,171 (34.3%)	5698 (34.1%)			4080 (29.4%)	17,461 (32%)	37,813 (32.3%)	
		Unknown	33,281 (27.7%)	15,597 (19.7%)	2491 (14.9%)			3541 (25.5%)	10,621 (19.5%)	16,960 (14.5%)	
healthy_diet_status		Mean ± SD	4.1 ± 1.1	4.2 ± 1.1	4.3 ± 1.1		< 0.001	3.4 ± 1.2	3.5 ± 1.2	3.6 ± 1.2	< 0.001
Cardioprotective_diet		Mean ± SD	3.4 ± 1.2	3.6 ± 1.2	3.7 ± 1.2		< 0.001	2.7 ± 1.2	2.8 ± 1.2	2.9 ± 1.2	< 0.001

### Gene-environment interaction analyses

In gene-environment analyses, we observed that diabetic status attenuated the protective effect of high RHGS PRS in abdominal hernia (RERI = −0.10 [CI −0.17, −0.04]; AP = −0.07 [−0.11, −0.02]), diaphragmatic hernia (RERI = −0.12 [CI −0.20, −0.04]; AP = −0.07 [−0.12, −0.02]), and diverticular intestine (RERI = −0.09 [CI −0.16, −0.01]; AP = −0.05 [−0.10, −0.01]). Likewise, hyperlipidemia conferred a consistent pattern of risk amplification in abdominal hernia (RERI = −0.08 [CI −0.13, −0.03]; AP = −0.04 [−0.07, −0.02]), diaphragmatic hernia (RERI = −0.09 [CI −0.15, −0.02]; AP = −0.05 [−0.08, −0.01]), but not in diverticular intestine (RERI = −0.03 [CI −0.09, 0.02]; AP = −0.02 [−0.05, 0.01]). Current smoking status similarly diminished the protective effect of high RHGS PRS in abdominal hernia (RERI = −0.04 [CI −0.07, 0]; AP = −0.03 [−0.06, 0]) but not in diaphragmatic hernia (RERI = −0.02 [CI −0.06, 0.03]; AP = −0.01 [−0.05, 0.02]), and diverticular intestine (RERI = −0.02 [CI −0.09, 0.04]; AP = −0.02 [−0.07, 0.03]). In contrast, a cardioprotective dietary pattern synergistically enhanced the protective effect of high RHGS PRS, reducing disease risk in abdominal hernia (RERI = 0.04 [CI 0.01, 0.08]; AP = 0.06 [0.01, 0.12]), diaphragmatic hernia (RERI = 0.04 [CI 0, 0.08]; AP = 0.05 [0, 0.10]), and diverticular intestine (RERI = 0.04 [CI 0, 0.09]; AP = 0.05 [0, 0.10]). Notably, total fiber intake demonstrated a unique synergistic protective effect specifically for diverticular intestine (RERI = 0.04 [CI 0.01, 0.08]; AP = 0.05 [0.01, 0.09]). Beyond these reported interactions, none of the remaining environmental factors yielded significance. In the split-sample validation, the gene-environment interaction with diabetic status proved robust: among the 30% independent test set, diabetic status again attenuated the protective effect of high RHGS PRS on diaphragmatic hernia (RERI = −0.08 [−0.14, −0.02]; AP = −0.07 [−0.13, −0.02]) and diverticular intestine (RERI = −0.08 [−0.16, −0.01]; AP = −0.03 [−0.05, −0.01]). Though lost statistical significance, the directions of effect for the PRS main term and the majority of G×E coefficients remained consistent with those observed in the full sample.

## Discussion

Our GWAS identified 226 genomic loci and 407 protein-coding genes associated with RHGS. Integration with skeletal muscle eQTLs via TWAS and colocalization highlighted biologically plausible candidates, including *L3MBTL3*, *CEP192* and *NUCKS1*. Mendelian randomization and polygenic-risk-score analyses revealed that genetically higher RHGS confers a protective effect against abdominal-wall hernia, diaphragmatic hernia, diverticular disease, NAFLD and peptic ulcer in a linear dose–response fashion. Finally, gene-environment interaction analyses demonstrated that cardioprotective diets and higher fiber intake amplify, whereas diabetes, hyperlipidemia and smoking attenuate, this protective relationship.

Skeletal-muscle eQTL-informed colocalization and TWAS singled out RHGS-associated variants that converge on genes repeatedly linked to muscle mass, strength and quality, positioning them at the functional core of muscle biology. *CCND2*, which encodes cell cycle protein D2, plays an essential role in cell cycle regulation by promoting the transition of cells from G1 phase to S phase, thereby affecting cell proliferation and growth. In skeletal muscle, the expression of *CCND2* may be associated with the proliferation, differentiation and regeneration of muscle cells [23, 24]. *MYH7* (myosin heavy chain 7) is expressed in slow-twitch muscle fibers and is one of the key proteins involved in muscle contraction. The expression level and function of *MYH7* are closely related to muscle strength and endurance. Mutations in *MYH7* can lead to muscle dysfunction, such as weakness and atrophy [25, 26]. *CA3*, is enriched in slow-twitch type I fibers, making it ideal for studying muscle fiber type shifting and adaptations. *CA3* levels differ in skeletal muscle tissues and biofluids in neuromuscular diseases, and its changes in age-related muscle wasting and dystrophinopathy make it a promising biomarker for the assessment of muscular function [27]. *ZBTB38*, also known as *PPP1R171*, is related to *PPP1R3A* and interacts with the glycogen-related PP1 heterodimer in skeletal muscle. It plays a crucial role in initiating the myogenic program [28]. The rs7624084 locus in *ZBTB38* is associated with low grip strength, and its mutation may reduce *ZBTB38* expression in skeletal muscle [29]. *L3MBTL3* is positioned within the MEF2/SRF transcriptional network that governs myogenesis and mitochondrial energetics [30], while *DLG1* scaffolds nNOS-GSNOR complexes during C2C12 differentiation, thereby tuning contractile signaling [31]. *NUCKS1*, a cell-cycle gatekeeper, synchronizes myoblast exit from the cell cycle by modulating cyclin-dependent cascades [32]. HOXB3 couples Wnt/Notch/β-catenin signaling to proliferation of mesenchymal precursors [33, 34]. *CEP192* ensures accurate centrosome-mediated spindle formation required for satellite-cell expansion [35–37]. The Golgi protein GOLGA1 maintains secretory flux for extracellular-matrix remodeling. Actin-binding *PDLIM4* [38] and MAP3K3-mediated p38/ERK activation integrate cytoskeletal dynamics with calcium sensitization, whereas calcium-buffer PEF1 and nuclear-pore component NUP160 fine-tune excitation-contraction coupling and nucleocytoplasmic transport [39–42]. Finally, microtubule-severing KATNA1 and asparaginyl-tRNA synthetase DARS sustain sarcomere assembly and nascent-protein synthesis, completing a molecular framework in which RHGS-associated variants may calibrate muscle mass, contractile quality and, consequently, gastrointestinal health [43, 44]. These genes sketch a coherent mechanistic perspective: alleles that curtail muscle mass or contractile efficiency simultaneously blunt the muscle’s endocrine and mechanical support of the gut. Our findings integrate these muscle-centric loci with the established “muscle-gut axis.” By amplifying systemic IL-6/TNF-α, compromising insulin-mediated collagen cross-linking, and depleting creatine-driven enteric-neuro-muscular signaling, genetically-determined low RHGS converts sarcopenia into the triad of leaky barrier, weak intestinal wall and high intraluminal pressure-precisely the pathogenic sequence we postulate for hernia and diverticula formation [45]. This genomics-guided framework offers a fresh perspective on muscle–gut comorbidity and positions RHGS as a measurable sentinel of digestive vulnerability.

The correlation and causality between low grip strength and systemic disorders is of particular importance in disease primary prevention. One study found that grip strength is significantly related to arterial stiffness and central arterial stiffness may mediate the relationship between grip strength and major cardiovascular events [46]. These findings are also supported by MR studies, which show that low grip strength causally associated with the increased risk of coronary heart disease, myocardial infarction, heart failure, hypertension, and stroke [47]. Prospective cohort and MR analysis also confirmed the positive correlation between low grip strength and diabetes risk [48, 49]. Post-GWAS studies indicated significant genetic correlation and causal association between Fried Frailty Score and cardiovascular-, neurological-, and inflammation-related diseases/traits [50]. Our GWAS and post-GWAS results show a widespread genetic negative correlation between RHGS and nine major disease categories, confirming that reduced RHGS (after BMI adjustment) systemically increase the risk of full spectrum of disorders.

Grip strength is increasingly viewed as a surrogate of systemic muscle quantity and quality. In older adults the correlation between grip strength and total-body muscle mass is especially tight in men, and the value of a handgrip dynamometer as a rapid proxy for overall strength has been formally validated [4, 51]. Because muscle loss tracks with declining bone mineral density, low grip strength predicts osteoporosis and incident fractures in women and parallels skeletal muscle wasting in heart-failure patients, underscoring its utility for monitoring age-related catabolism [52, 53]. Beyond locomotion and metabolism, adequate muscle tone is essential for the structural integrity of body-wall compartments. Controlled trials show that pharmacological inhibition of abdominal-wall muscle contraction enlarges and multiplies incisional hernias, whereas contraction limits defect size, implying that stronger abdominal musculature, reflected in higher grip strength, protects against hernia formation. Similar principles apply to the diaphragm [54]. Grip strength correlates positively with vital capacity, FEV₁ and maximal expiratory pressure in stroke survivors and with diaphragm thickness in systemic sclerosis [55, 56]. In ventilated patients, weaker grip predicts extubation failure and, when combined with diaphragm ultrasound, improves the diagnosis of ICU-acquired weakness [57, 58]. Collectively, these data indicate that peripheral muscle strength and diaphragmatic performance decline in parallel, making grip strength a convenient window into respiratory muscle reserve. Intestinal diverticular disease is another condition in which muscle assessment is informative. Because diverticulosis is linked to malnutrition and sarcopenia, a grip-strength index adjusted for BMI complements traditional nutritional markers and supplies clinicians with integrated information on both energy stores and functional muscle mass [59]. Taken together, the converging epidemiological and interventional evidence positions grip strength as a translatable, low-cost biomarker that connects systemic muscle status with the pathophysiology of abdominal-wall hernia, diaphragmatic dysfunction and colonic diverticula, thereby providing a rationale for muscle-targeted preventive strategies in gastrointestinal practice.

Our own GWAS and post-GWAS findings provide genetic corroboration for these epidemiological parallels. By demonstrating that 1,111 independent SNPs influence RHGS and that a one-standard-deviation increase in the RHGS polygenic risk score lowers odds of abdominal hernia, diaphragmatic hernia and diverticular disease, we show that the muscle digestive axis is at least partly genetically determined. Our Mendelian randomization provided genetic evidence consistent with a possible causal relationship, contingent on the standard MR assumptions. A genetically predicted one-unit rise in RHGS conferred lower risk of abdominal hernia, diaphragmatic hernia and diverticular intestine, effects that persisted after adjustment for BMI and other confounders. Because 180 of the prioritized genes are highly expressed in type I/II myocytes and mesenchymal cells, the protective signal appears to originate from skeletal muscle itself rather than from generalized body size.

Importantly, we also clarify that diabetes, hyperlipidemia and smoking attenuate the protective effect of a high RHGS PRS, whereas cardioprotective diet and greater fiber intake enhance it, mirrors of the environmental modifiers previously described in grip-strength cohort studies. These are in line with previous findings, showing that smoking or healthy dietary patterns can alleviate oxidative stress, promote the colonization of normal gut microbiota and maintain the integrity of intestinal epithelium and mucosal immunity [60, 61]. Thus, our genetic data validate the clinical intuition that stronger muscle is protective, quantify the magnitude of benefit, and identify modifiable amplifiers that clinicians can target. By embedding RHGS polygenic screening into routine risk assessment, gastroenterologists and surgeons could stratify patients for muscle-building exercise programs, peri-operative conditioning, or intensified metabolic management, thereby converting a simple grip-dynamometer reading into a genetically informed, actionable intervention for gastrointestinal health. However, we should note that while the attenuation of RHGS PRS protection by diabetes reproduced directionally in the independent 30% subset, we emphasize that this G×E finding remains hypothesis-generating. The validation sample was only one-third the size of the discovery set, so estimates are imprecise and compatible with both weaker and stronger interactions; formal replication in external cohorts with comparable diabetes definitions and hernia phenotyping is therefore essential before any causal or clinical inference is drawn.

Our observation that diabetes, hyperlipidemia and smoking may blunt the protective effect of a high RHGS PRS, while cardioprotective diet and higher fiber intake might amplify it, mirrors previous grip-strength reports [60, 61]. Quantifying these modifiers offers clinicians a genetically anchored argument to promote smoking cessation, peri-operative glycemic control and muscle-building exercise in patients whose inherited grip-muscle advantage is otherwise eroded by metabolic insults. Nevertheless, we should note that while the attenuation of RHGS PRS protection by diabetes reproduced directionally in the independent 30% subset, we emphasize that this gene-environment interaction finding remains hypothesis-generating as many of the tests lost significance in splitting populations and the further validations in external cohorts are warranted.

Our study has several limitations. First, the GWAS was restricted to European-ancestry participants; replication and trans-ethnic meta-analyses are required to broaden the applicability of the findings. Second, although we adopted a 70/30 split-sample strategy to curb over-fitting, all GWAS, PRS construction and disease/interaction tests still originate from the same parent cohort, and independent external replication is currently lacking. Consequently, the generalizability of our PRS and interaction estimates to non-European or other population settings remains to be established, and future studies should seek validation in publicly accessible biobanks with comparably deep phenotyping and genomic data. Third, because RHGS may share genetic architecture with metabolic traits, multi-trait approaches (e.g., MTAG) could reveal additional associated variants. Fourth, our regression results indicated a robust but epidemiologically small effects on the risk of disease. This might be due to the fact that genotype imputation does not capture the full allele-frequency spectrum; rare coding and regulatory variants remain to be systematically evaluated through whole-genome and exome sequencing. Finally, the clinical associations require confirmation in independent cohorts or randomized trials, and functional studies are needed to clarify how the implicated gene-environment interactions modulate digestive disease risk.

## Conclusion

In summary, our genome-wide analysis identified multiple genetic loci and candidate genes associated with RHGS, many of which are functionally active in skeletal muscle tissue. Genetically predicted RHGS showed significant inverse associations with a broad spectrum of digestive disorders, including abdominal hernia, diaphragmatic hernia and diverticular disease. Our findings support the polygenic risk score for RHGS may aid in individual risk stratification and clinical surveillance, while lifestyle factors such as diet and comorbidities modulate these genetic effects.

## Supplementary Information

Supplementary Material 1: Supplementary figure 1. Tissue specificity and enrichment analyses A and B. Bar plots depicting tissue-wise enrichment of prioritized genes using deTS (A) and FUMA (B). C. Enrichment of genomic regions using rGreat. D. Enrichment of key biological functions using DisGeNET. E. Enrichment analyses using gprofiler. FUMA, functional mapping and annotation of genetic associations. Supplementary figure 2. Association of polygenic risk score of RHGS on the risk of duodenal ulcer, gastric ulcer and peptic ulcer using regression models and restrictive cubic spline analyses. A and B. Heatmap showing the logistic regression (A) and Cox regression (B) results of the polygenic risk score of RHGS (RHGS PRS) on duodenal ulcer, gastric ulcer and peptic ulcer. The model 1: raw model without adjustment (model 1); the model 2: multi-adjusted model with common covariates including age, sex, BMI, townsend deprivation index, smoking status, alcohol consumption, education, ethnicity and albumin levels; the model 3: with further adjustment of urate levels, diabetes, and hyperlipidemia on top of the model 2; the model 4: further adjusted model with physical activity and waist-to-height ratio on top of the model 3; the model 5: further adjusted model with cardioprotective diet on top of the model 4. C-E. Restrictive cubic spline illustrating the potential linearity between RHGS PRS and duodenal ulcer (C), gastric ulcer (D), and peptic ulcer (E) risks using logistic regression. F-H. Restrictive cubic spline illustrating the potential linearity between RHGS PRS and duodenal ulcer (F), gastric ulcer (G), and peptic ulcer (H) risks using Cox regression. OR: odds ratio; HR: hazards ratio; CI: confidence interval. Supplementary figure 3. Association of polygenic risk score of RHGS on the risk of major liver disorders using regression models and restrictive cubic spline analyses. A and B. Heatmap showing the logistic regression (A) and Cox regression (B) results of the polygenic risk score of RHGS (RHGS PRS) on ALD, NAFLD, liver fibrosis, ESLD, and liver cancer. The model 1: raw model without adjustment (model 1); the model 2: multi-adjusted model with common covariates including age, sex, BMI, townsend deprivation index, smoking status, alcohol consumption, education, ethnicity and albumin levels; the model 3: with further adjustment of urate levels, diabetes, and hyperlipidemia on top of the model 2; the model 4: further adjusted model with physical activity and waist-to-height ratio on top of the model 3; the model 5: further adjusted model with cardioprotective diet on top of the model 4. C-G. Restrictive cubic spline illustrating the potential linearity between RHGS PRS and ALD (C), NAFLD (D), liver fibrosis (E), ESLD (F), and liver cancer (G) risks using logistic regression. H-L. Restrictive cubic spline illustrating the potential linearity between RHGS PRS and ALD (H), NAFLD (I), liver fibrosis (J), ESLD (K), and liver cancer (L) risks using Cox regression. ALD: alcoholic fatty liver disease; NAFLD: non-alcoholic fatty liver disease; ESLD: end-stage liver disease; OR: odds ratio; HR: hazards ratio; CI: confidence interval. Supplementary figure 4. Association of RHGS measures on the risk of abdominal hernia, diaphragmatic hernia, diverticular intestine, duodenal ulcer, gastric ulcer, and peptic ulcer using regression models. A and B. Heatmap showing the logistic regression (A) and Cox regression (B) results of the measure of RHGS on abdominal hernia, diaphragmatic hernia, and diverticular intestine. C and D. Heatmap showing the logistic regression (C) and Cox regression (D) results of the measure of RHGS on duodenal ulcer, gastric ulcer, and peptic ulcer. The model 1: raw model without adjustment (model 1); the model 2: multi-adjusted model with common covariates including age, sex, BMI, townsend deprivation index, smoking status, alcohol consumption, education, ethnicity and albumin levels; the model 3: with further adjustment of urate levels, diabetes, and hyperlipidemia on top of the model 2; the model 4: further adjusted model with physical activity and waist-to-height ratio on top of the model 3; the model 5: further adjusted model with cardioprotective diet on top of the model 4. OR: odds ratio; HR: hazards ratio; CI: confidence interval. Supplementary figure 5. Sensitivity analyses with split samples for GWAS analyses and PRS results A. Manhattan plot for the *p-values* of all variants. The purple dash line indicates the genome-wide significance level of 5 × 10^−8^. A’. Q-Q plot for the GWAS analyses. B and C. Heatmap showing the logistic regression (B) and Cox regression (C) results of the polygenic risk score of RHGS (RHGS PRS) on abdominal hernia, diaphragmatic hernia and diverticular intestine in the non-overlapping 30% overall population. The model 1: raw model without adjustment (model 1); the model 2: multi-adjusted model with common covariates including age, sex, BMI, townsend deprivation index, smoking status, alcohol consumption, education, ethnicity and albumin levels; the model 3: with further adjustment of urate levels, diabetes, and hyperlipidemia on top of the model 2; the model 4: further adjusted model with physical activity and waist-to-height ratio on top of the model 3; the model 5: further adjusted model with cardioprotective diet on top of the model 4. RHGS: relative hand grip strength. GWAS: genome-wide association study.

Supplementary Material 2: Supplementary table 1. ICD9 and ICD10 codes of diseases in the study. Supplementary table 2. Genomic risk loci table from FUMA. Supplementary table 3. Independent significant SNPs. Supplementary table 4. Lead SNP table. Supplementary table 5. Mapped gene tables (from FUMA). Supplementary table 6. ANNOVAR results. Supplementary table 7. Enrichment analysis using rGreat. Supplementary table 8. Enrichment analysis using gprofiler. Supplementary table 9. Tissue specificity analyses using deTS. Supplementary table 10. Colocalization. Supplementary table 11. TWAS (PrediXcan) results in the skeletal muscle. Supplementary table 12. TWAS (JTI) results in the skeletal muscle. Supplementary table 13. FOCUS credible genes (PIP > 0.9). Supplementary table 14. LDSC genetic correlation. Supplementary table 15. Mendelian randomization using inverse variance weighted (IVW) method. Supplementary table 16. Mendelian randomization using weighted median method. Supplementary table 17. Mendelian randomization using MR-Egger method. Supplementary table 18. Pleiotropic analyses in Mendelian randomization. Supplementary table 19. Heterogeneity analyses in Mendelian randomization. Supplementary table 20. Logistic regression of RHGS PRS on the disease risks. Supplementary table 21. Cox regression of RHGS PRS on the disease risks. Supplementary table 22. Logistic regression of RHGS measures on the disease risks. Supplementary table 23. Cox regression of RHGS measure on the disease risks. Supplementary table 24. Logistic regression of RHGS measures on the disease risks in the rest of 30% population. Supplementary table 25. Cox regression of RHGS measures on the disease risks in the rest of 30% population.

Supplementary Material 3.

## Data Availability

Genome-wide summary statistics are available from the FinnGen database upon publication. Individual-level UK Biobank data can be accessed by approved applicants at https://www.ukbiobank.ac.uk/. Supplementary materials are deposited in the OSF repository (https://osf.io/uj4fq/).
