# Supplementary figures and images for: Genome-wide association and integrative analyses of relative handgrip strength identify polygenic determinants of gastrointestinal disorder susceptibility
Source: BMC Gastroenterol. 2026 Jan 27;26:138. doi: 10.1186/s12876-026-04624-9 (PMC12918038; doi:10.1186/s12876-026-04624-9)

**Supplementary figures**

**SP1.**

**
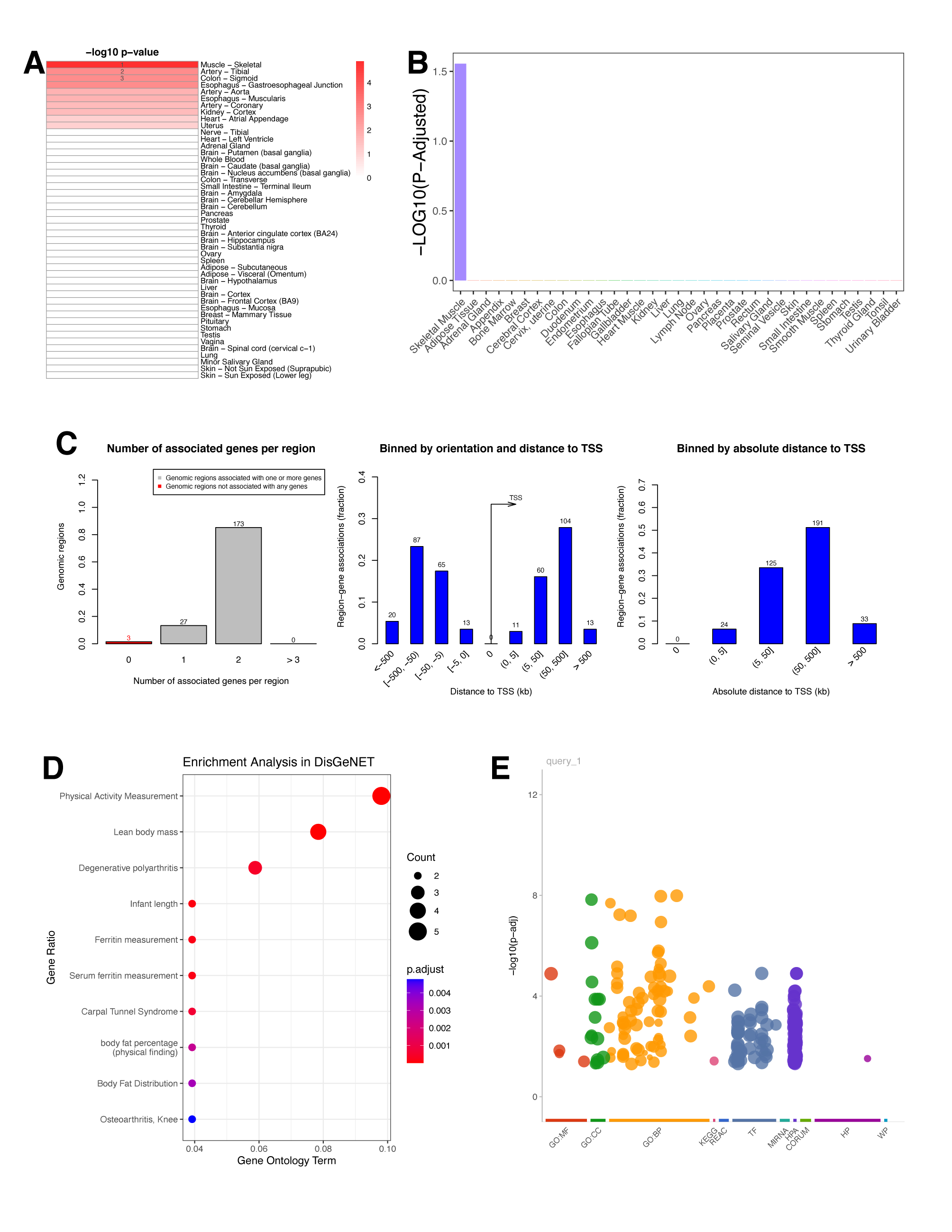
**

**SP2.**

**
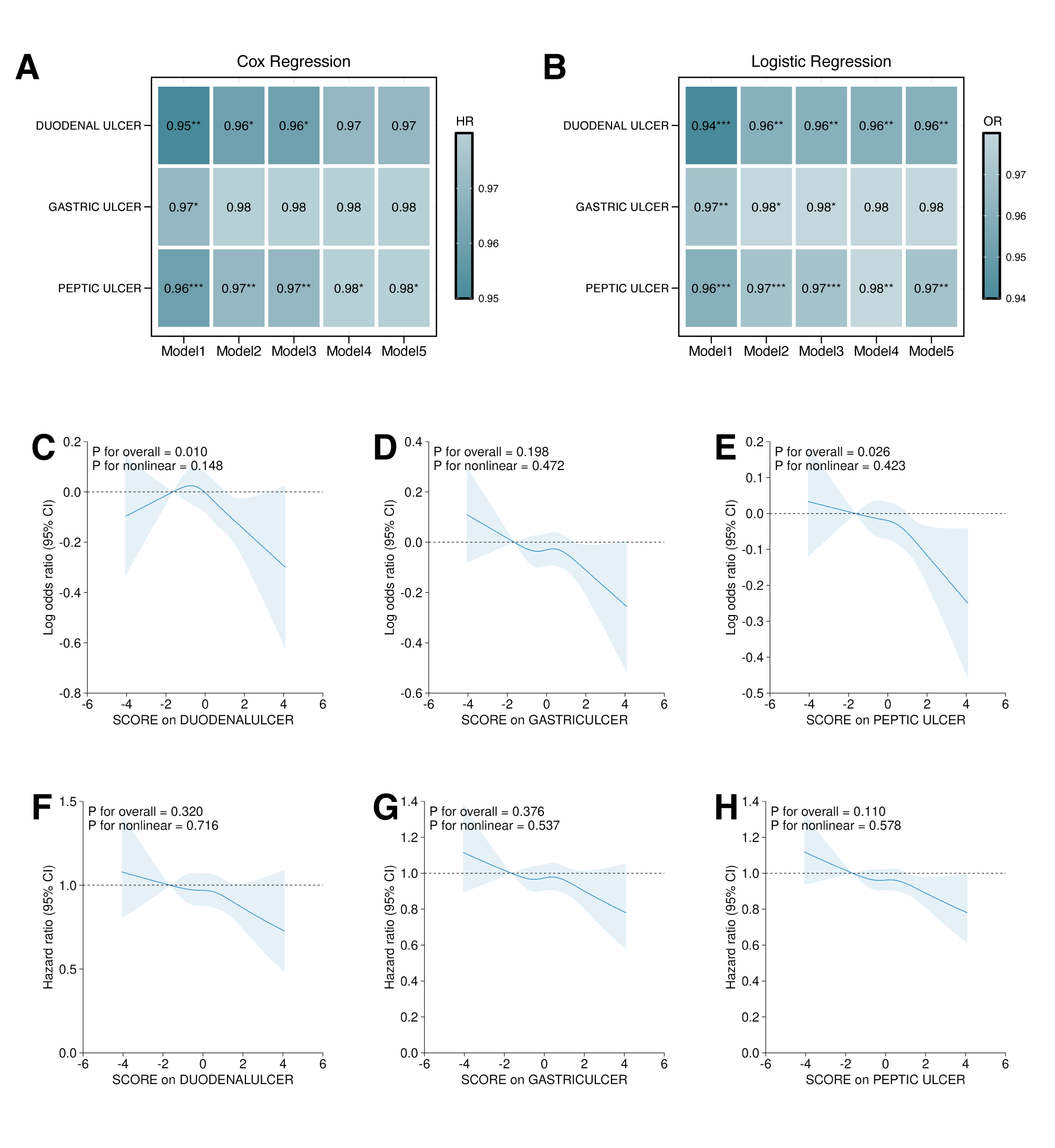
**

**SP3.**

**
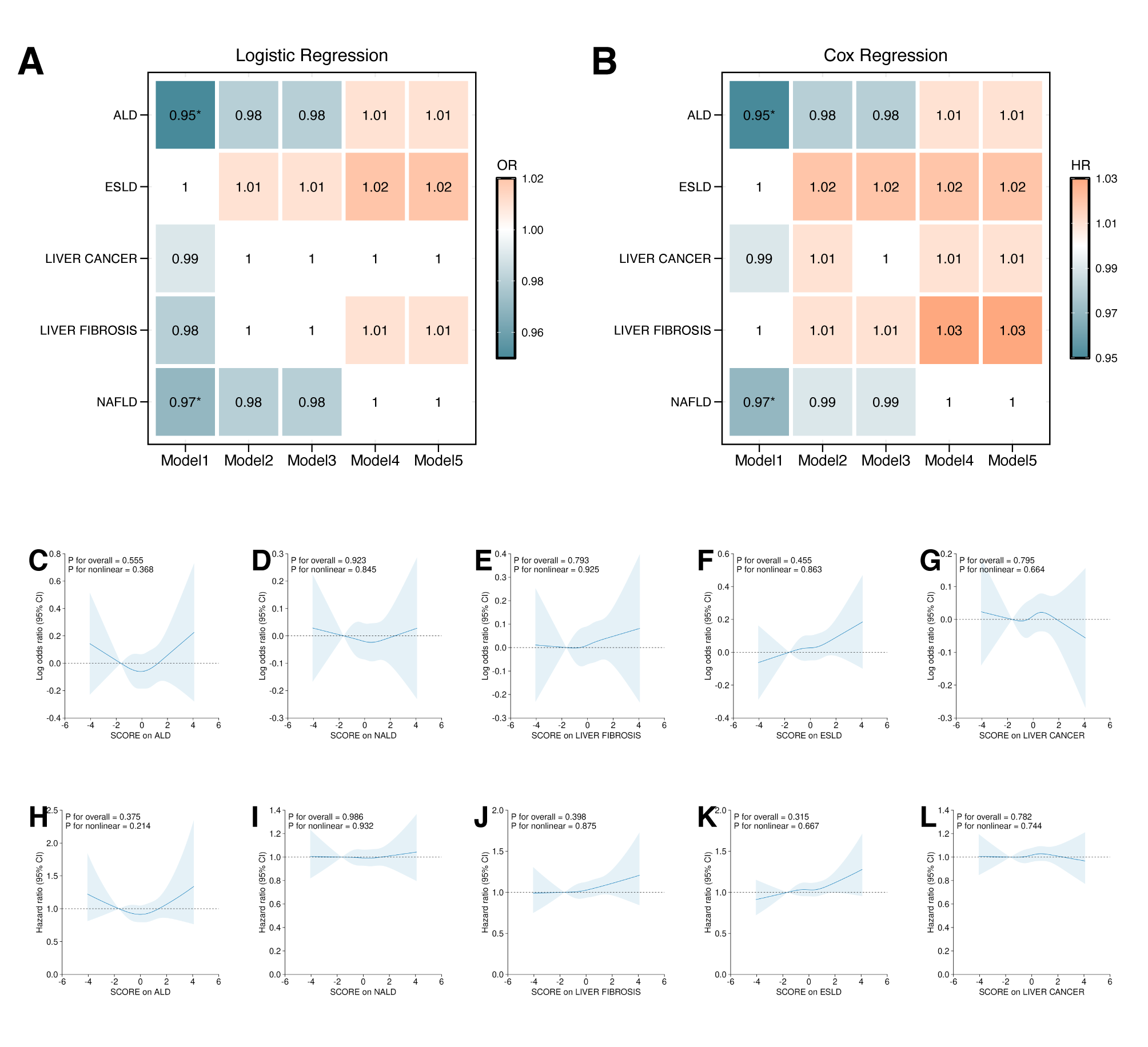
**

**SP4.**

**
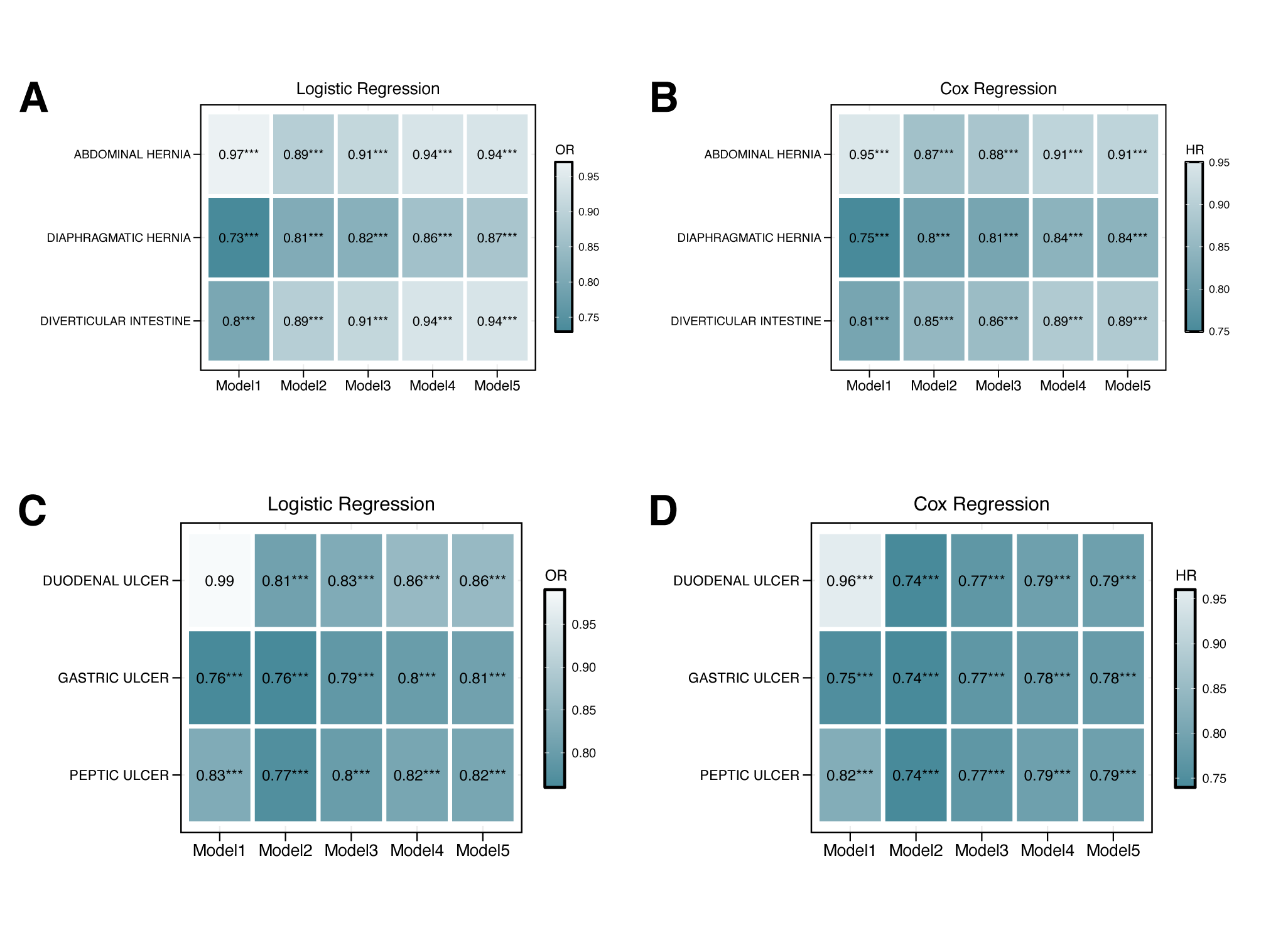
**

**SP5.**

**
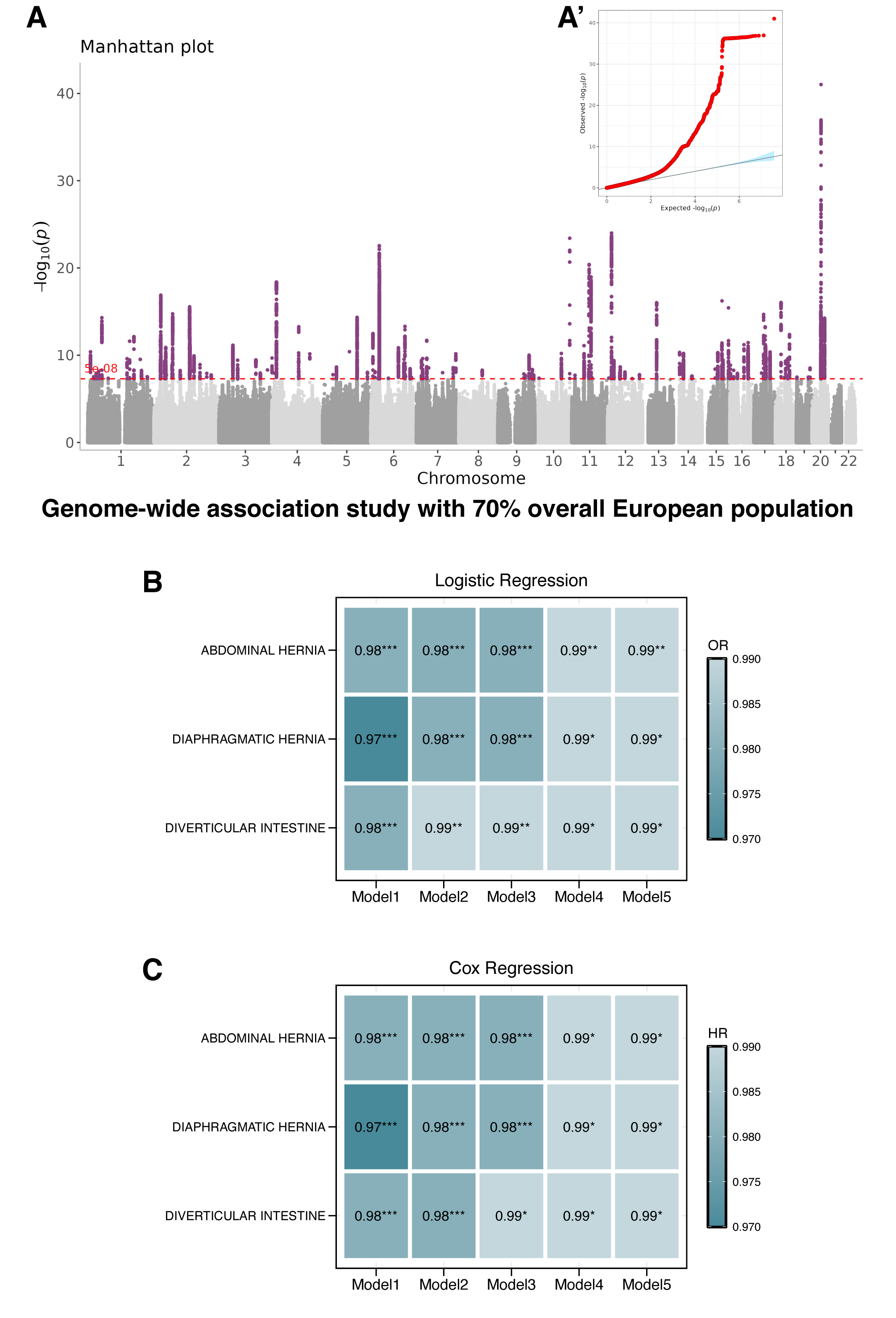
**

Supplement: Supplementary file 1 — Supplementary Material 1: Supplementary figure 1. Tissue specificity and enrichment analyses A and B. Bar plots depicting tissue-wise enrichment of prioritized genes using deTS (A) and FUMA (B). C. Enrichment of genomic regions using rGreat. D. Enrichment of key biological functions using DisGeNET. E. Enrichment analyses using gprofiler. FUMA, functional mapping and annotation of genetic associations. Supplementary figure 2. Association of polygenic risk score of RHGS on the risk of duodenal ulcer, gastric ulcer and peptic ulcer using regression models and restrictive cubic spline analyses. A and B. Heatmap showing the logistic regression (A) and Cox regression (B) results of the polygenic risk score of RHGS (RHGS PRS) on duodenal ulcer, gastric ulcer and peptic ulcer. The model 1: raw model without adjustment (model 1); the model 2: multi-adjusted model with common covariates including age, sex, BMI, townsend deprivation index, smoking status, alcohol consumption, education, ethnicity and albumin levels; the model 3: with further adjustment of urate levels, diabetes, and hyperlipidemia on top of the model 2; the model 4: further adjusted model with physical activity and waist-to-height ratio on top of the model 3; the model 5: further adjusted model with cardioprotective diet on top of the model 4. C-E. Restrictive cubic spline illustrating the potential linearity between RHGS PRS and duodenal ulcer (C), gastric ulcer (D), and peptic ulcer (E) risks using logistic regression. F-H. Restrictive cubic spline illustrating the potential linearity between RHGS PRS and duodenal ulcer (F), gastric ulcer (G), and peptic ulcer (H) risks using Cox regression. OR: odds ratio; HR: hazards ratio; CI: confidence interval. Supplementary figure 3. Association of polygenic risk score of RHGS on the risk of major liver disorders using regression models and restrictive cubic spline analyses. A and B. Heatmap showing the logistic regression (A) and Cox regression (B) results of the pol [file 12876_2026_4624_MOESM1_ESM.docx]
